# Outcomes and patterns of use of Radium-223 in metastatic castration-resistant prostate cancer

**DOI:** 10.3389/fonc.2024.1385466

**Published:** 2024-05-07

**Authors:** Urbano Anido-Herranz, Ovidio Fernandez-Calvo, Juan Ruiz-Bañobre, Sara Martinez-Breijo, Natalia Fernandez-Nuñez, Zulema Nogareda-Seoane, Miguel Garrido-Pumar, Javier Casas-Nebra, Gloria Muñiz-Garcia, Paula Portela-Pereira, Antonio Gomez-Caamaño, Daniel Adolfo Perez-Fentes, Lucia Santome-Couto, Martín Lázaro, Aurea Molina-Diaz, Ana Medina-Colmenero, Sergio Vazquez-Estevez

**Affiliations:** ^1^ Translational Medical Oncology Group (ONCOMET), Health Research Institute of Santiago de Compostela (IDIS), University Clinical Hospital of Santiago de Compostela, University of Santiago de Compostela (USC), Santiago de Compostela, Spain; ^2^ Department of Medical Oncology, University Clinical Hospital of Santiago de Compostela (SERGAS), University of Santiago de Compostela (USC), Santiago de Compostela, Spain; ^3^ Department of Medical Oncology, University Clinical Hospital of Ourense, Ourense, Spain; ^4^ Centro de Investigación Biomédica en Red de Cáncer (CIBERONC), Instituto de Salud Carlos III, Madrid, Spain; ^5^ Department of Urology, University Clinical Hospital of A Coruña, A Coruña, Spain; ^6^ Department of Medical Oncology, Lucus Augusti University Hospital, Lugo, Spain; ^7^ Department of Nuclear Medicine, University Clinical Hospital of Santiago de Compostela, Santiago de Compostela, Spain; ^8^ Department of Urology, Lucus Augusti University Hospital, Lugo, Spain; ^9^ Department of Nuclear Medicine – GALARIA, Complexo Hospitalario Universitario Ourense A. S. de Ourense, Ourense, Spain; ^10^ Department of Urology, Area Sanitaria de Ourense, Ourense, Spain; ^11^ Department of Radiation Oncology, University Clinical Hospital of Santiago de Compostela, Santiago de Compostela, Spain; ^12^ Department of Urology, EOXI University Clinical Hospital of Santiago de Compostela, Santiago de Compostela, Spain; ^13^ Department of Medical Oncology, POVISA, Vigo, Spain; ^14^ Department of Medical Oncology, Álvaro Cunqueiro Hospital, Vigo, Spain; ^15^ Department of Medical Oncology, University Clinical Hospital of A Coruña, A Coruña, Spain; ^16^ Department of Medical Oncology, Fundación Centro Oncológico de Galicia, A Coruña, Spain

**Keywords:** metastatic castration-resistant prostate cancer, Radium-223, alkaline phosphatase, bone health agents, real-world data

## Abstract

**Introduction:**

Radium-223 dichloride (Ra-223) is recommended as a treatment option for metastatic castration-resistant prostate cancer (mCRPC) patients with symptomatic bone metastases and no visceral disease, after docetaxel failure, or in patients who are not candidates to receive it. In this study, we aimed to ambispectively analyze overall survival (OS) and prognostic features in mCRPC in patients receiving Ra-223 as per clinical routine practice and identify the most suitable treatment sequence.

**Patients and methods:**

This study is observational, multicentric, and ambispective. Eligibility criteria included mCRPC patients treated with Ra-223, with an Eastern Cooperative Oncology Group (ECOG) performance status of 0–2, without visceral metastases, and no more than three cm involved lymph nodes.

**Results:**

A total of 145 patients were included; the median age was 73.97 years, and a Gleason score of more than or equal to 7 in 61 (48%) patients; 73 (81%) had previously received docetaxel. The most important benefit was reached by those patients who received Ra-223 in the second-line setting, with a median OS of 17 months (95% CI, 12–21), and by patients who received six cycles of treatment, with a median OS of 19 months (95% CI, 14–21). An alkaline phosphatase (ALP) decrease was also identified as a prognosis marker. When performing the multivariate analysis, the time to develop castration-resistant disease longer than 24 months was the most important prognostic factor to predict the evolution of the patients receiving Ra-223. Ra-223 was well tolerated, with thrombocytopenia, anemia, and diarrhea being the main adverse events.

**Conclusion:**

There is a benefit for those patients who received Ra-223 in the second-line setting, regardless of prior use of docetaxel. In addition, a survival benefit for patients presenting with a decline in ALP was observed.

## 1 Introduction

Prostate cancer is the second most common tumor in men worldwide, and its incidence has been increasing over the last decades in most countries, especially in Asia and Northern and Western Europe (1). However, mortality rates have been reduced, mainly due to early diagnosis approaches and treatment improvements (2). It is a common disease, impacting importantly in the healthcare system (3).

The treatment landscape for metastatic castration-resistant prostate cancer (mCRPC) has been evolving dramatically in recent years. The first approved chemotherapy showing a benefit in overall survival (OS) was docetaxel in 2004 (4). Thereafter, the number of available approved agents has been increasing, but the therapeutic sequence is based on clinical characteristics. Due to this quick increase in the number of available treatment choices, data supporting treatment sequences is lacking still, and guidelines only provide support based on nonrandomized clinical trials for treatment sequencing (5).

Radionuclides are treatments that have been widely used for prostate cancer. Ra-223 is a targeted alpha emitter that selectively binds to areas of increased bone turnover in bone metastases and emits high-energy alpha particles of short-range (< 100 µm) (6).

Ra-223 was the first radiopharmaceutical to achieve benefit in OS and time to the first symptomatic skeletal-related event (SRE) in patients with mCRPC and bone metastases (5). This was assessed in a phase 3, double-blinded clinical trial (ALSYMPCA) (7, 8). The eligibility criteria included patients with two or more symptomatic bone metastases, no visceral disease, and previous docetaxel failure (or patients not being candidates for it). Patients were randomized 2:1 to receive Ra-223 at a dose of 50 KBq/kg or placebo every 4 weeks for six doses. The primary objective was OS, and patients were stratified by ALP (< 220 IU/L versus ≥ 220 IU/L), concomitant use of bisphosphonates, and previous treatment with docetaxel. Ra-223 reduced the risk of death by 30% with a hazard ratio (HR) of 0.70 (95% CI, 0.58–0.83; *p* < 0.001), maintaining a consistent benefit in all analyzed subgroups. In addition, it reduced the risk of developing a symptomatic SRE by 34% with a HR of 0.66 (95% CI, 0.52–0.83) (5, 8). Regarding “patient-reported outcomes” (PROs) (7), Ra-223 was associated with better results than placebo, with slower deterioration based on EQ-5D questionnaires for the general population and Functional Assessment of Cancer Therapy-Prostate (FACT-P) for patients with prostate cancer, and a very good toxicity profile in addition to being well tolerated.

There are other treatments that may be administered in the mCRPC setting, as non-bone targeted therapies. Enzalutamide is an antiandrogenic drug that was tested in a phase 3 trial AFFIRM (9), that randomized 1,199 mCRPC patients to enzalutamide versus placebo, showing a clear OS benefit for enzalutamide of 18.4 months (95% CI, 17.3–not reached) versus 13.6 months (95% CI, 11.3–15.8) (HR, 0.63; 95% CI, 0.53–0.75; *p* < 0.001). Enzalutamide also improved quality of life and radiographic progression-free survival (PFS) (9, 10). After the AFFIRM study results, enzalutamide was evaluated in the pre-chemotherapy setting, with the PREVAIL trial comparing enzalutamide versus placebo in mCRPC patients. Radiographic PFS was also significantly better for enzalutamide (65 versus 14%, HR, 0.19; 95% CI, 0.15–0.23; *p* < 0.001), with an OS HR 0.71 (95% CI, 0.60–0.84; *p* < 0.001) (11).

Abiraterone acetate is a CYP17 inhibitor that showed positive results through several randomized trials. COU-AA-301 compared abiraterone acetate and prednisone (AAP) versus placebo in patients who had already progressed to chemotherapy (docetaxel). The median OS was 14.8 months for AAP versus 10.9 months for placebo, HR 0.65 (95% CI, 0.54–0.77; *p* < 0.001). Radiographic PFS was 16.5 versus 8.3 months, HR 0.53 (95% CI, 0.45–0.62; *p* < 0.001) (12, 13). Thereafter, the COU-AA-302 trial (14), a phase 3 study placebo-controlled in 1,088 asymptomatic or mildly symptomatic patients with chemotherapy-naive mCRPC, showed that median OS was significantly longer in the AAP than in the placebo arm (34.7 months versus 30.3 months; HR, 0.81; 95% CI, 0.70–0.93; *p* = 0.0033). In both studies, the safety profile was acceptable.

Both docetaxel and cabazitaxel have shown benefits in OS in mCRPC patients. The TAX327 study compared docetaxel-prednisone versus mitoxantrone, in a three-arm trial, testing docetaxel with two different schedules: every week (30 mg/m^2^) and every 3 weeks (75 mg/m^2^). Docetaxel being administered with the 3-week posology was superior in terms of OS with a HR of 0.76 (95% CI, 0.62–0.94; *p* = 0.009), with a median OS of 16.5 months for mitoxantrone, 18.9 months for 3-weekly, and 17.4 months for weekly docetaxel (4). With these results, 3-weekly docetaxel became the standard of care.

Cabazitaxel is a new-generation taxane that was compared to mitoxantrone in the phase 3 TROPIC study (15), which included patients who had developed progressive disease on docetaxel. Cabazitaxel reached a median OS of 15.1 months (95% CI, 14.1–16.3 months) versus 12.7 months (95% CI, 11.6–13.7 months) for mitoxantrone (HR, 0.70; 95% CI, 0.59–0.83; *p* < 0.0001). This trial made cabazitaxel a new standard of care after progression to docetaxel. Cabazitaxel was also directly compared to docetaxel in the phase 3 FIRSTANA study (16), including an additional arm with a different dose of cabazitaxel. The median OS was 24.5 months for cabazitaxel with a dose of 20 mg/m^2^, 25.2 months with a cabazitaxel dose of 25 mg/m^2^, and 24.3 months for docetaxel. Cabazitaxel did not prove to be better than docetaxel in terms of OS; hence, its indication remained as a second line after progression to docetaxel.

Patients with deoxyribonucleic acid (DNA) repair damage may benefit from poli-adenosine-diphosphate-ribose polymerase (PARP) inhibitors such as olaparib, niraparib, and talazoparib. Olaparib was tested in a phase 3 study (PROFOUND), including patients who had previously received one novel hormonal therapy line (either AAP or enzalutamide) and harbored a mutation in a DNA reparation gene (17). Patients were randomized to olaparib versus physician’s choice (AAP or enzalutamide), and the primary objective was radiographic PFS, which was dramatically longer for olaparib (7.4 versus 3.6 months; HR, 0.34; 95% CI, 0.25–0.47; *p* < 0.001) (17). Later on, the PROPEL trial met its primary endpoint, showing statistically significant improvement in radiographic progression-free survival with olaparib plus AAP versus placebo plus AAP in patients with first-line mCRPC unselected by homologous recombination repair mutation (HRRm) status (18, 19). In addition, the MAGNITUDE trial found patients with HRRm, particularly BRCA1/2, benefit from first-line therapy with niraparib plus AAP. Patients with mCRPC were prospectively identified as HRRm with/without BRCA1/2 alterations and randomized 1:1 to niraparib (200 mg orally) plus AAP (1000 mg/10 mg orally) or placebo plus AAP. In the second interim analysis, niraparib plus AAP significantly prolonged radiographic PFS, with median radiographic PFS 19.5 versus 10.9 months; HR 0.55 (95% CI, 0.39–0.78) (20). The TALAPRO-2 trial is a randomized, double-blind, phase 3 trial of talazoparib plus enzalutamide versus placebo plus enzalutamide as first-line therapy in men with asymptomatic or mildly symptomatic mCRPC receiving ongoing androgen deprivation therapy. Patients were prospectively assessed for HRR gene alterations in tumor tissue and randomly assigned to talazoparib 0.5 mg or placebo, plus enzalutamide 160 mg, administered orally once daily. Randomization was stratified by HRR gene alteration status and previous treatment with life-prolonging therapy in the castration-sensitive setting. The primary endpoint was radiographic PFS, evaluated in the intention-to-treat population, and at the planned primary analysis, median radiographic PFS was not reached (95% CI, 27.5 months–not reached) for talazoparib plus enzalutamide and 21.9 months (16.6–25.1) for placebo plus enzalutamide HR 0.63 (95% CI, 0.51–0.78; *p* < 0.0001) (21).

We present an observational study aiming to present data on OS in real-world populations, including mCRPC patients treated with Ra-223 in seven Spanish hospitals, assessing prognostic and analytic factors to select those patients who may get the highest benefit, besides identifying the best treatment sequencing, including Ra-223 in these patients.

## 2 Materials and methods

A multicenter, epidemiological, postauthorization, ambispective study was performed; 89.36% of patients were collected retrospectively and 37.59% after the publication of the ERA-223 study. A median follow-up of 11 months was obtained from 145 patients from seven Galician centers between March 2013 and December 2022. All patients signed an informed consent form accepting to participate in this observational study.

All consecutive patients who were going to start or were already receiving Ra-223 matching the eligibility criteria were included. Patients’ background and treatment outcomes, including prostate-specific antigen (PSA) decline, analytic parameters, biochemical and radiographic PFS, OS, and adverse events (AEs), were investigated.

Patients were scheduled to receive the Ra-223 administered dose as per labeling guidelines, six injections of Radium-223 (at a dose of 50 kBq/kg of body weight intravenously), and one injection was administered every 4 weeks, following the standard-of-care approach. Disease progression was defined according to the Prostate Cancer Working Group version 2 (PCWG2) criteria (22) for biochemical and radiographic PFS. OS was defined as the time from the first dose of Ra-223 to death. AEs were assessed according to the National Cancer Institute Common Terminology Criteria for Adverse Events version 4.0.

The statistical analysis was made with R Project (R Core Team (2018). R: A language and environment for statistical computing. R Foundation for Statistical Computing, Vienna, Austria. URL https://www.R-project.org/) and R Studio for Mac. Data analysis was performed using the following R packages: “gt”, “survminer”, “readxl”, “ggsignif”, “survival”, “survivalAnalysis”, “tidyverse”, “rstatix”, “gtsummary”, and “rms”. Missing data were evaluated with “naniar”, “visdat”, “VIM”, “rpart”, and “finalfit” packages, eliminating variables with more than 30% of missing data. There was no loss of follow-up to the principal outcomes (biochemical and radiographic PFS, OS). At the final analysis cutoff date, only 11 patients remained alive.

For independent sample analyses, Pearson’s Chi-square statistical test or Fisher’s exact test was used for qualitative variables, and the Student’s *t*-test, ANOVA, or its nonparametric equivalents, Mann–Whitney *U* and Kruskal–Wallis *H* in the case of quantitative variables.

Biochemical and radiographic PFS and OS were determined from Kaplan–Meyer survival curves; Cox regression models were used to determine predictive/associated factors for OS. In order to determine the factors associated with the general response rate, a logistic regression model was carried out, whose dependent variable was the response, and as independent variables all those possible factors that were significant in the univariate model. The analysis population included all patients who had received at least one Radium-223 injection and had data entered into the study database at the time of the data cutoff. A minimal follow-up of 6 months after the last patient ended treatment was required before the cutoff date for data analysis.

The Sankey diagram was performed to assess the sequence of treatments and flow of patients, paying special attention to patients who died or continued on treatment.

The sponsor followed the development of the study in accordance with the requirements of the legislation applicable to clinical research and postauthorization studies, complying with the requirements contemplated in Law 14/2007 on Biomedical Research and Order SAS/3470/2009.

## 3 Results

Up to 145 patients were included, with a median follow-up of 11 months. Baseline characteristics are detailed in **Table 1**. The median age was 74 years, Gleason > 7 in 61 (48%) patients, and 66 (53%) patients had stage IV disease at first diagnosis; 73 (81%) had previously received docetaxel. Most patients, 64 (47%), received Ra-223 in the second-line setting. The most received treatment besides Ra-223 was docetaxel, with 46 (51%) in the first line and 20 (17%) out of them in hormone-sensitive disease. Regarding hormonal treatments, 60 (45%) had received AAP, 79 (59%) received enzalutamide, and only one patient received apalutamide. Six (4.4%) patients received Ra-223 as the only treatment received.

**Table 1:** Demographic characteristics for the entire population.

Characteristic	N = 145
Age (median; IQR)	74 (69–80)
Group age (*n*; %)	
≤ 70	50 (35)
> 70	91 (65)
Group Gleason (*n*; %)	
> 7	61 (48)
≤ 7	65 (52)
ECOG (*n*; %)	
0	35 (25)
1	93 (67)
2	11 (7.9)
Cycles of Radium (*n*; %)	
1	9 (6.5)
2	12 (8.6)
3	9 (6.5)
4	12 (8.6)
5	14 (10)
6	83 (60)
Hemoglobin (median; IQR)	12.25 (11.00–13.25)
Baseline PSA (median; IQR)	69 (15–214)
Baseline ALP (median; IQR)	205 (105–429)
Number of nonaxial mets (median; IQR)	2 (0–5)
Adenopathies (*n*; %)	30 (26)
Time to CRPC (months; median; IQR)	31 (15–76)
Use of abiraterone (*n*; %)	60 (45)

The development of castration-resistant disease in these patients took 31.2 months, or 11.33 months from the initial diagnosis to the development of metastases. Progressive disease was mainly radiographic in 56 (40%) patients.

Liver failure (any grade) was present in up to three (2.4%) patients, and five (3.9%) had renal failure. Up to 25 (20%) patients had diabetes mellitus.

The Eastern Cooperative Oncology Group (ECOG) performance status was 1 in 93 (67%) patients at the moment of starting Ra-223. Focusing on bone metastases, 38 (36%) did not have any extra-axial bone metastases, whereas 32 (30%) had five or more extra-axial bone metastases. Lymph nodes were present in 30 (26%) of patients, defined by the RECIST 1.1 criteria.

The median PSA before starting Ra-223 was 69 ng/mL (range, 0.05–6,222 ng/mL). The median hemoglobin was 12.25 g/dL, and 16 (13%) patients had required transfusions before starting Ra-223. The median lactate dehydrogenase (LDH) was 338 U/L. The median ALP was 205 UI/L. There were no statistically significant differences among Gleason scores based on ALP, PSA, LDH, and hemoglobin.

**Figure 1:**
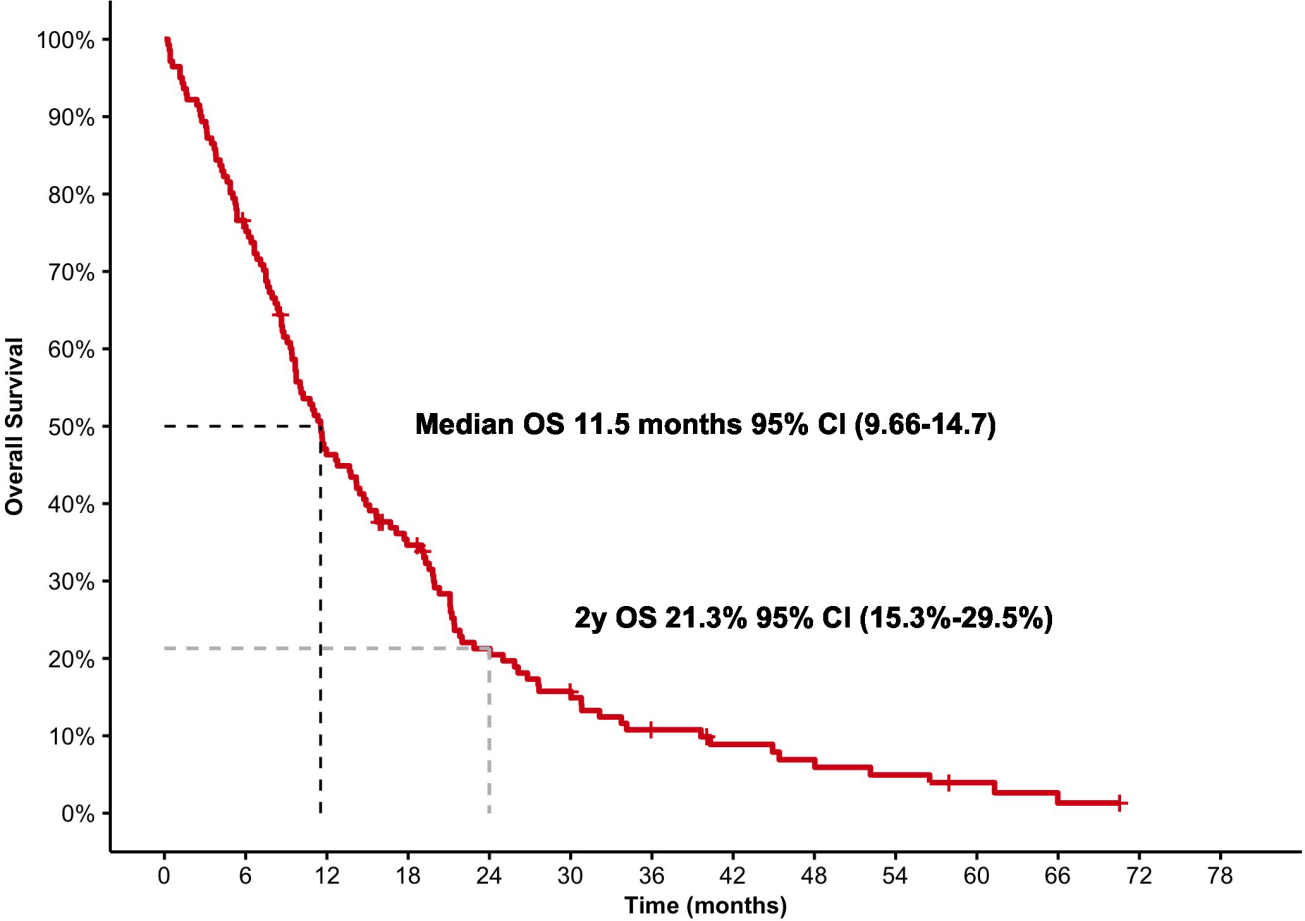
Overall survival for the entire population.

Median OS was 11.9 months (95% CI, 9.66–14.7) for the entire population, independent of the number of lines for Radium-223 (see **Figure 1**), with 46% (95% CI, 39%–55%) of patients alive 12 months after Ra-223 was started and 21% (95% CI, 15%–30%) at 24 months. Median OS was similar in terms of age at the beginning of Ra-223 (≤ 70 versus > 70 years) (HR, 1.14; 95% CI, 0.8–1.7; *p* = 0.46), with a median OS at 12 months, 11.8 months (95% CI, 9.70–15.1) for patients being at least 70 years versus 10.1 months (95% CI, 8.37–21.2) for patients being 70 or younger.

There were no differences in terms of Gleason score (≤ 7 versus > 7). There were no differences in survival in terms of liver insufficiency, diabetes mellitus, or patients with positive lymph nodes (*p* = 0.4). However, ECOG at the beginning of Ra-223 showed statistically significant differences (*p* < 0.001).

### 3.1 Treatment

In total, 83 (60%) patients completed six cycles of Ra-223. Most patients (98; 72%) received Ra-223 in the second or third line, although some patients received it in a very late setting: 27 patients (20%).

Those patients who received 6 cycles of Ra-223 reached a better OS, a median of 19.26 months (95% CI, 14.43–21.45) compared to those who received a shorter treatment, who had a median OS of 5.32 months (95% CI, 4.17–9.45) (HR, 0.27; 95% CI, 0.19–0.41; *p* < 0.0001).

Focusing on those patients who received Ra-223 in the first-, second-, or third-line setting versus those who received it beyond, there is a statistically significant difference in terms of OS (HR, 2.62; 95% CI, 1.27–5.42; *p* = 0.009). Patients who received Ra-223 in the second line reached the best results in terms of OS at 17 months (95% CI, 12–21; *p* = 0.017).

**Figure 2:**
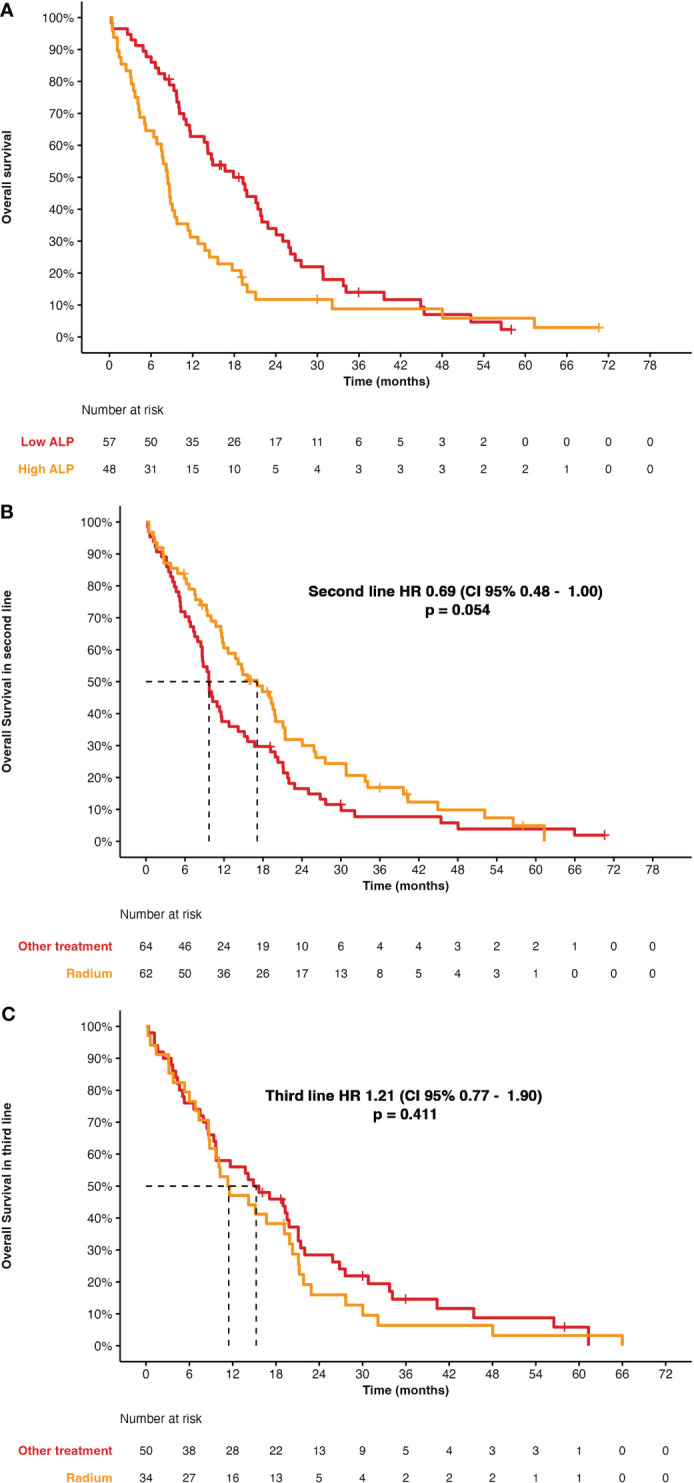
**(A)** Overall survival according to levels of ALP (cutoff, 220 UI/L). **(B)** Overall survival for Ra-223 in second-line versus other treatments. **(C)** Overall survival for Ra-223 in third-line versus other treatments.

ALP at baseline allowed the differentiation of two groups, based on whether ALP was ≤ 220 UI/L or > 220 UI/L. Patients with lower ALP had a better OS of 17.97 months (95% CI, 13.7–22.9) compared to 8.43 months (95% CI, 8.43–11.3) (HR, 0.46; 95% CI, 0.30–0.71; *p* = 0.0067) (see **Figure 2A**).

There were statistically significant differences among those patients who had or had not received previous docetaxel (HR, 1.97; 95% CI, 1.13–3.42; *p* = 0.016), with a median OS 21 months (95% CI, 20–57) for docetaxel after Ra-223 and 10 months (95% CI, 9.3–15) for docetaxel before Ra-223. There were no differences among patients who had received AAP before with a median OS of 9.9 months (95% CI, 8–21; *p* = 0.078) or after Ra-223 with a median OS of 22 months (95% CI, 9.7–NR) (HR, 1.90; 95% CI, 0.92–3.94; *p* = 0.083), but patients who were on enzalutamide before Ra-223 had a worse median OS of 9.7 months (95% CI, 8.6–14; *p* < 0.001) than afterward with a median OS of 34 months (95% CI, 31–NR) (HR, 3.93; 95% CI, 1.73–8.92; *p* = 0.001).

There were no differences in OS based on patients who had reached a biochemical response HR of 0.95 (95% CI, 0.81–1.12; *p* = 0.6). However, there was a clear benefit for patients who completed treatment, with a median OS of 19 months (95% CI, 14–21) compared with a median lower than 5.4 months (95% CI, 3.8–9.4) for those patients who had to discontinue due to progressive disease and those who achieved symptoms improvement (HR, 0.46; 95% CI, 0.28–0.76; *p* = 0.003).

Depending on whether patients developed clinical progression or not, there is also a statistically significant HR of 1.71 (95% CI, 1.12–2.61; *p* = 0.013) with a median OS of 20 months (95% CI, 14–26) for those patients who did not progress clinically, versus 9.7 months (95% CI, 8.4–14) for patients with clinical progression. These differences were not observed based on PSA response (*p* = 0.012).

However, despite there being no statistically significant differences, those patients who achieved a response in terms of ALP (53) had also a duration of treatment of approximately 5.48 months (most of them completing six cycles) and achieved the most important benefit in survival 22 months (95% CI, 20–48). Furthermore, the biochemical progression date is very close to the end of the treatment date, although two patients had biochemical progression and remained on treatment.

In our study, only 18 (15%) patients achieved a response in terms of PSA (decline PSA ≥ 30%). There was no difference in neutrophil/lymphocyte ratio, which did not change during treatment (*p* = 0.46).

Treatment duration for AAP was clearly better in the first or second line compared to the third or fourth line (*p* < 0.001), with a median of 11 months (95% CI, 8.3–19) in the first line, 9.4 months (95% CI, 5.5–NA) in the second line, 4 months (95% CI, 3.9–NA) in the third line, and 2.3 months (95% CI, 1.1–NA) in the fourth line.

Similar results are seen for enzalutamide without achieving statistically significant differences: 13 months (95% CI, 10–19) in the first line, 9.2 months (95% CI, 4.2–NA) in the second line, 4.4 months (95% CI, 2.6–NA) in the third line, and 7.0 months (95% CI, 6.2–NA) in the fourth line.

Cabazitaxel did not have differences in duration of treatment based on the line setting (*p* = 0.4); however, there was only one patient receiving cabazitaxel as first-line treatment, who received Ra-223 in a very late line. The median OS for those patients who received it in the second line was 5.1 months (95% CI, 3.7–NA).

Ra-223 was compared against other treatments by line of treatment, showing no differences between them (HR, 0.69; 95% CI, 0.48–1.00; *p* = 0.054), with a median OS of 17.2 months (95% CI, 8.64–12.8) for Ra-223. In the third line, there were also no differences (HR, 1.21; 95% CI, 0.77–1.90; *p* = 0.411), with a median OS for Ra-223 of 11.4 months (95% CI, 8.80–20.3) (see **Figures 2B, C**).

A multivariate analysis was done, including age, Ra-223 treatment line, number of cycles, ALP levels, and hemoglobin levels.

Regarding the treatment line, the later Ra-223 is administered, the higher the risk of death is, considering that, for those patients who receive Ra-223 in a very late line, there is an additional negative bias since those patients have a poorer prognosis and clinical progressive disease may impact on the reason for discontinuation.

A period to develop castration-resistant disease longer than 12 months shows no differences as predictive factor (HR, 1.22; 95% CI, 0.65–2.30).

The number of received Ra-223 cycles (completion of treatment) is also a clear good prognosis factor, with an HR of 0.27 (95% CI, 0.19–0.41; *p* < 0.0001). ALP levels of more than 220 UI/L increase the risk of death (HR, 0.46; 95% CI, 0.30–0.71; *p* = 0.0067). See **Table 2** for univariate and multivariate analyses.

**Table 2:** Univariate and multivariate analysis.

	Univariate Analysis			Multivariate Analysis		
Characteristic	N	HR (95% CI)^1^	p–value	HR (95% CI)^1^	p–value	Adjusted p–value^2^
**Grouped Age**	141					
≤70		—		—		
>70		1.15 (0.80 to 1.66)	0.46	0.77 (0.48 to 1.24)	0.28	0.723
**Alkaline Phosphatase**	105					
≤220 U/L		—		—		
>220 U/L		1.75 (1.16 to 2.63)	0.007	1.15 (0.69 to 1.91)	0.60	0.723
**Cycles of Radium**	139	0.28 (0.19 to 0.41)	<0.001	0.23 (0.13 to 0.40)	<0.001	0.005
**Hemoglobin**	108					
>9.5 g/dL		—		—		
≤9.5 g/dL		2.81 (1.39 to 5.67)	0.004	0.99 (0.35 to 2.75)	0.98	0.987
**Radium–223 in 2^nd^ line**	126					
Other treatment		—		—		
Radium		0.70 (0.48 to 1.01)	0.055	0.59 (0.36 to 0.97)	0.040	0.048

**Figure 3:**
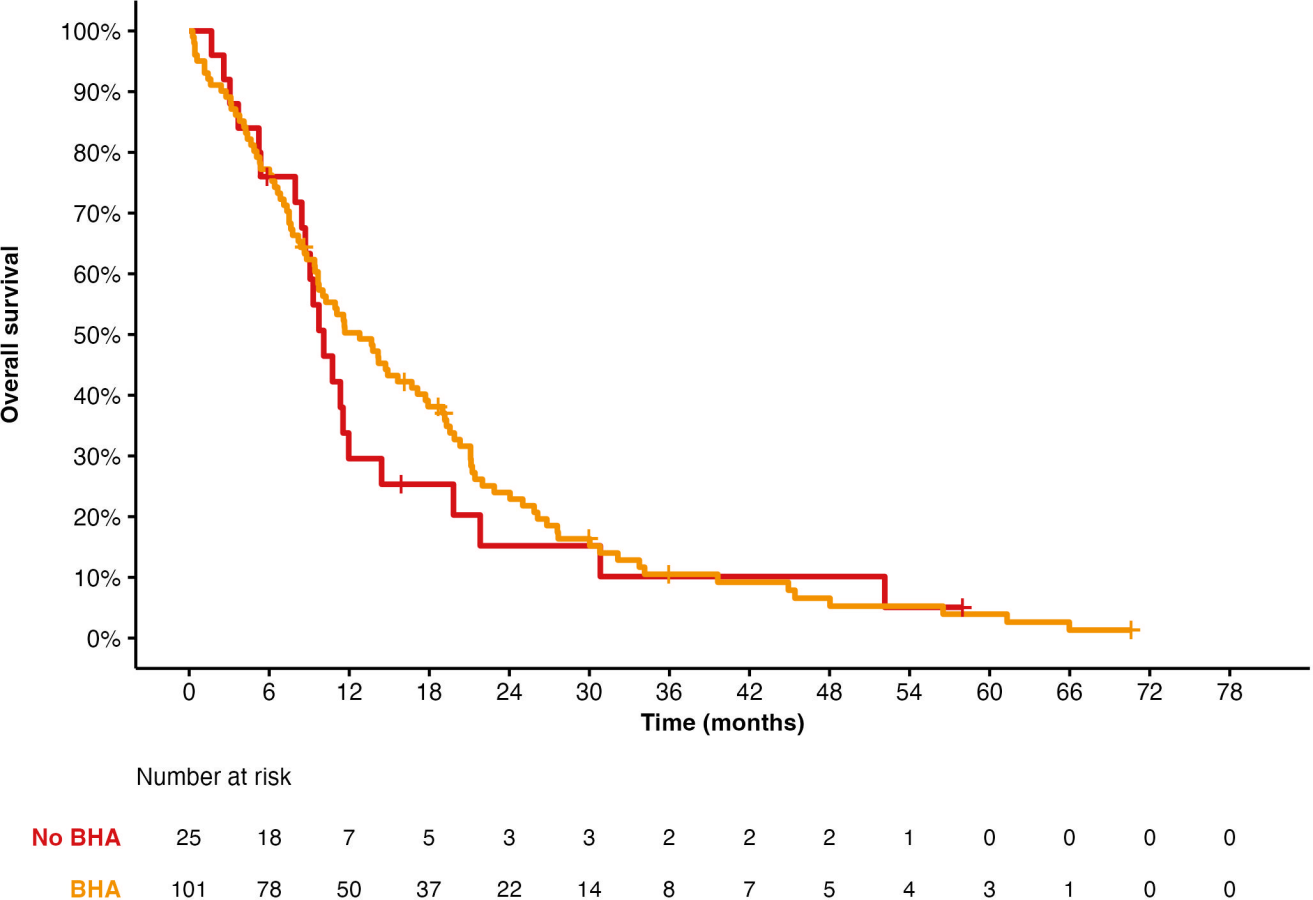
Overall survival for Ra-223 based on having received bone health agents (BHA) or not.

Up to 80% of patients received bisphosphonate therapy (zoledronic acid or denosumab). There was no statistically significant difference among patients who had or had not received a bone-targeted therapy (HR, 0.87; 95% CI, 0.54–1.39; *p* = 0.056), with a median OS 12.8 months (95% CI, 8.74–19.8) and 10.1 months (95% CI, 8.7–19.8) for bone-targeted therapy-treated patients versus nonbone-targeted therapy-treated patients (see **Figure 3**).

Among those patients who received Ra-223 in the second or third line, there were no statistically significant differences in terms of Gleason, age, previous PSA, type of progression, or number of received Ra-223 cycles.

**Figure 4:**
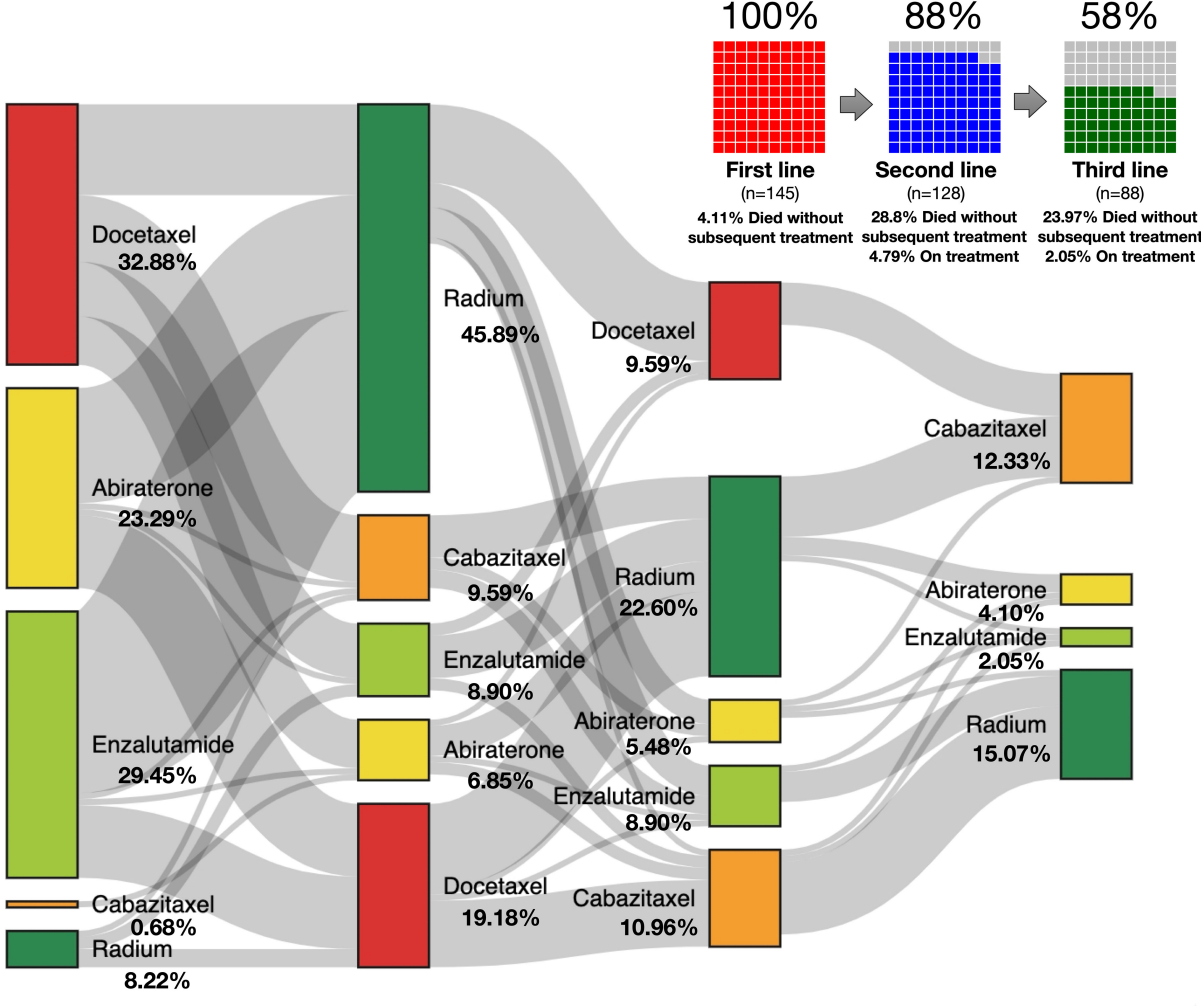
Sankey diagram showing patients’ flow chart with the percentage of patients who did not receive a subsequent line.

There was no preferred treatment after Ra-223; however, Ra-223 was more commonly administered after docetaxel and much less commonly administered after enzalutamide or AAP (see **Figure 4**).

### 3.2 Safety

Ra-223 is a well-tolerated treatment. In our sample, we observed 20% grades 1–2 thrombocytopenia (2.8% grade 3) and 78% grades 1–2 anemia (11% grade 3). Anemia is much higher than expected compared to the available literature. At least one-third of patients were treated with Ra-223 in the third line or beyond, and this may be the cause of the greater frequency and grade of anemia in this series. Our recommendation to control this toxicity is to avoid Ra-223 treatment with levels of hemoglobin below 9.5 g/dL or in patients with bone marrow involvement by tumor based on clinical trial inclusion/exclusion criteria; 6.7% of patients had grade 1–2 diarrhea, which is consistent with the literature. We did not find a statistically significant difference in OS for those patients who had developed any grade of thrombocytopenia compared to those who did not present it. Hemoglobin levels showed differences in the univariate analysis (HR, 2.81; 1.39–5.67; *p* = 0.004), but in the multivariate analysis, there were no differences (HR, 0.99; 0.35–2.75; *p* = 0.98).

## 4 Discussion

The strength of this study was the broad eligibility criteria that encompassed all mCRPC patients treated with Ra-223, whatever the treatment line. Our analysis has, in addition, a few limitations, including its ambispective nature and the lack of sample size calculation. However, our results are mostly aligned with prior available literature.

The pivotal study that led to approval for Ra-223 was the ALSYMPCA trial (8), where the median OS was 14.9 months for patients receiving Ra-223 compared to 11.3 months for the control arm (HR, 0.7; 95% CI, 0.58–0.83; *p* < 0.001). In a subgroup analysis, patients who received biphosphonates (HR, 0.70; 95% CI, 0.52–0.93) and those who did not receive them (HR, 0.74; 95% CI, 0.59–0.92) reached benefit from Ra-223; patients who received treatment with docetaxel prior (HR, 0.71; 95% CI, 0.56–0.89) or after Ra-223 (HR, 0.74; 95% CI, 0.56–0.99) had similar benefits.

Other studies have assessed OS in patients receiving Ra-223. Álvarez Pérez et al. (23) reported a median OS of 11 months (95% CI, 9.95–12.04); Carles et al. (24) found a median OS of 14 months (95% CI, 10–NA), which is more aligned with the ALSYMPCA results. Additionally, the early access study showed a median OS of 20.5 months (95% CI, 20.5–NA) versus 13.5 months (95% CI, 11.7–17.1) and a HR of 0.48 (95% CI, 0.32–0.72), including even asymptomatic patients (25).

Moreover, the rechallenge study (26) achieved a median OS of 24 months, with 78% and 50% at 12 and 24 months, respectively.

A more recent ERa-223 study has been shared (27), which included patients with mCRPC and at least two or more bone metastases who are asymptomatic or mildly symptomatic, with an ECOG performance status of 0–1, without visceral or brain metastases, and without any prior chemotherapy, who were randomized to AAP with Ra-223 versus AAP-placebo. This study was closed earlier than expected, as there was an increase in mortality in the experimental arm. In November 2017, HR for OS was 1.347 (95% CI, 1.04–1.73; *p* = 0.02) with a median OS for experimental of 30.7 months (95% CI, 25.2–35.6) versus 33.3 months (95% CI, 30.2–NA) for patients on AAP-placebo. Additionally, there was a higher number of bone fractures for the experimental arm (26% versus 8.1%). With these data, the study was closed and the EMA summary of product characteristics for Ra-223 was updated, limiting its use to those patients who had received at least two prior systemic treatment lines or who were not candidates for any systemic therapy. However, with a longer follow-up, HR for OS was 1.195 (95% CI, 0.95–1.50; *p* = 0.12); hence, OS was similar for both arms (28).

In our study, the median OS was 12 months (95% CI, 9.7–15), with 46% (95% CI, 39–55) of patients alive 12 months after Ra-223 was started and 21% (95% CI, 15–30) at 12 and 24 months. This is slightly lower than those results showed at ALSYMPCA, probably due to being a nonselected population, with many patients receiving Ra-223 in a late stage. As with most cancer treatments, the median OS decreases the later the line in which it is used, so comparing between the second or third line, there are statistically significant differences in a HR of 2.62 (95% CI, 1.27–5.42; *p* = 0.009) with better outcomes in those patients being treated in the second line, with a median OS of 17 months (95% CI, 12–21; *p* < 0.001), whereas for those patients who receive Ra-223 in the third line or beyond, median OS was 11 months (95% CI, 8.8–20) and, for those patients in the fourth or further line, the median OS is 7.5 months (95% CI, 5.1–9.7).

Hence, it could be suggested that the second or third line would be the most recommended setting for Ra-223, regardless of whether patients had received prior docetaxel or not (29), as this does not impact the outcomes.

The Stattin et al. study (30) found a moderately increased mortality risk in patients treated with Ra-223 in the first line, which was not observed in later lines of treatment as in this series. This result, as they claim, should be interpreted with caution since confounding is possible. For example, patients receiving Ra-223 as first line were likely ineligible for other treatments, perhaps due to frailty or comorbidities.

Regarding the sequencing data, there is nothing yet published; as previously explained, prior docetaxel does not impact the effectiveness of Ra-223, and although Ra-223 results are clearly better in the second line, where they reach a higher median OS than other treatments with statistically significant differences (*p* < 0.001), in the third-line setting, median OS decreases a little but no significant differences were seen when comparing Ra-223 with the rest of the treatments (*p* < 0.001) (25).

In the exploratory analysis of ALP (31) of the ALSYMPCA study, levels of ALP, PSA, and LDH were associated with OS in the intention-to-treat population. ALP decreased at 12 weeks in 87% of patients significantly compared to placebo (23%; *p* < 0.001), LDH decreased by 51% compared to 34% (*p* = 0.003), and PSA decreased by 27% and 14%, respectively (*p* = 0.160). In the case of patients with decreased ALP, this led to a 55% decrease in the risk of death in a HR of 0.45 (95% CI, 0.34–0.61), with a proportional effect of treatment for ALP as a surrogate for OS of 0.34 (95% CI, 0–0.74). However, ALP did not meet the statistical requirements necessary to be considered a surrogate for overall survival. More recently, Romero-Laorden et al. (32) performed a prospective cohort study of patients treated with Ra-223. They found that biomarkers of bone formation, especially ALP, have prognostic value in these patients.

Other smaller studies have analyzed this parameter: Uemura et al. studied efficacy and safety in 19 patients, with 54.5% achieving a decrease in ALP (33). Wenter et al. (34) treated 10 patients, with a median decrease in ALP of 28%. Álvarez Pérez et al. treated 68 patients with a 68% decrease of at least 30% in ALP (23). An early access study published by Carles et al. had 60% ALP responses with at least a 30% decrease (24).

In our study, we found significant differences with a cutoff point of 263 IU/L. Patients with lower ALP had a median OS of 18 months (95% CI, 14–23) versus 8.3 months (95% CI, 5.2–11) for higher ALP, with a HR for risk of death of 1.52 (95% CI, 0.84–2.76). Although only 19 of 53 patients showed a decrease in ALP greater than 30%, the Kaplan–Meier did not show differences in survival between patients who presented response, stabilization, or increase in this parameter. However, the patients who presented a decrease higher than 30% had a median OS of 18 months (95% CI, 14–23), slightly more than double compared to patients who had an increase in ALP, with a median OS of 8.3 months (95% CI, 5.2–11). Thus, although we have not found statistically significant differences among ALP levels or their change during treatment, these data are in line with what has been published by other authors, and ALP seems to be a better prognostic biomarker than PSA for Ra-223 treatment.

In the ALSYMPCA study (8), PSA exploratory analyses were done showing a clear relation between baseline level and OS (*p* ≤ 0.0003), with a proportional effect in PSA change at 12 weeks as a survival surrogate of 0.07 (95% CI, 0–0.21). In this study, differences in PSA responses were not seen between Ra-223 versus placebo, although PSA increase occurred in 99 (85%) of the patients at 12 weeks without correlation between PSA and OS in the multivariate analysis. Thus, it did not meet the statistical requirements to consider it a surrogate for OS, but it does serve to monitor the disease (31).

In other studies, PSA showed practically no responses. In the study by Uemura et al. (33), there was only a PSA reduction of ≥ 30% in two patients out of 16, with none of them reducing the PSA by ≥ 50%. In the study by Álvarez Pérez (23), a PSA decrease of ≥ 30% was reported in 23.9% of patients, with an increase in PSA in 60.6% of patients. In the study by Carles et al. (24), a percentage of PSA responses of ≥ 30% in 16% was seen, being ≥ 50% in 9% of the patients. In the international early access program, a percentage of PSA responses occurred in 21% of asymptomatic patients and 13% in symptomatic patients.

In our study, the biochemical response was collected without specifying a threshold change percentage, and no differences in terms of OS were seen in a HR of 0.67 (95% CI, 0.40–1.14; *p* = 0.14), although only 18 (15%) patients achieved a biochemical response.

In 2011, one of the first analyses was published regarding time to resistance to castration as a prognostic factor for survival (35), with an HR of 0.66 (95% CI, 0.57–0.87; *p* = 0.003) in the multivariate analyses for patients who had received mainly docetaxel or other chemotherapies and had a time to castration resistance of less than 2 years. This prognostic factor was also confirmed later for other drugs, such as enzalutamide (36, 37) and AAP (36, 38), but it was not confirmed as an independent predictive factor for survival. However, there are some studies in which time to castration resistance was shown to be an independent prognostic factor for OS and a predictor of response to docetaxel, such as the study by Suer et al. (39) where 162 patients treated with docetaxel were recruited, with a median time to castration resistance of 18 months and a HR for OS of 2.8 (95% CI, 1.92–3.28; *p* = 0.001) and a HR for response to docetaxel of 2.2 (95% CI, 1.15–4.08; *p* = 0.001).

Our data showed that patients who had a time to castration resistance of fewer than 12 months had a median OS of 9.1 months (95% CI, 5.3–21), compared to 13 months (95% CI, 10–18) for patients who had 12 months or more to develop castration resistance (*p* = 0.7).

### 4.1 Number of cycles

In the ALSYMPCA study, patients who received between one and four cycles had shorter survival on a *post-hoc* analysis (40). The median OS for patients who received one to four cycles was 6.2 months and 6.3 months in the early access study, whereas patients receiving five to six cycles of Ra-223 had a longer OS (*p* < 0.0001). The phase 3b study by Heidenreich et al. (41) observed similar data to those published by Sartor et al. in the expanded access performed in the USA (42) where patients receiving between one and four cycles had a median OS of 7.5 months (95% CI, 6.5–8.2), compared with patients receiving between five and six cycles who, at the time of publication of the study, had not yet reached it. In this study, patients receiving five to six cycles of Ra-223 had better prognosis profiles based on a lower burden of metastatic disease, a lower percentage of these patients had received prior docetaxel, less severe baseline bone pain, a lower percentage of patients with an ECOG-PS2, and lower PSA and ALP values. It was even observed that, in most cases, the reason for discontinuing Ra-223 treatment was disease progression.

In our study, the number of received cycles was also clinically significant, with an HR of 0.30 (95% CI, 0.17–0.52; *p* < 0.001) in favor of patients who received six cycles compared to those who received fewer, with a median of 19 months (95% CI, 14–21) for those who completed the treatment and 5.4 months (95% CI, 3.8–9.4) for those who did not. Furthermore, in the multivariate analysis, the number of received cycles remained significant with an HR of 0.61 (95% CI, 0.52–0.71). This is in line with all the published literature, but it is also likely to have a survival bias.

### 4.2 Safety and skeletal-related events

The ERA 223 study (27) was closed prematurely due to an increased risk of death and a higher percentage of fractures among patients who received Ra-223, probably related to poor bone health. However, with a longer follow-up and more events, survival was equal between both groups.

Regarding bone fractures and SREs, in our study, there were four pathological fractures (nonosteoporotic) and three spinal cord compressions (one of them at diagnosis), and this is probably related to the wide use of bone-directed therapies in these patients (80% of patients).

On the other hand, the REASSURE study (43) found 1% of second malignancies with a similar median follow-up and 1,465 patients, and other toxicities were consistent with previous clinical trials. We did not find cases of second malignancies, but this association requires further and cautious investigations.

## 5 Conclusions

Prostate cancer has had new approved treatments, and the landscape has been changing rapidly. Ra-223 is a well-tolerated treatment with a good safety profile; SREs may be controlled by administering bone-targeted therapies. It is key to select those patients who could get the most important benefit.

Improvement in ALP value has been identified as a prognosis factor in patients receiving Ra-223. However, by itself, it does not impact patients’ outcomes, and other factors must be considered.

Ra-223 achieves the best results when administered in a second-line setting, regardless of prior use of docetaxel. Other important prognostic factors include baseline PSA, time to develop castration-resistant disease, and the number of received Ra-223 cycles (despite survival bias).

## Data availability statement

The raw data supporting the conclusions of this article will be made available by the authors, without undue reservation.

## Ethics statement

The study was approved by the Galician Research Ethics Committee and conducted in accordance with the guidelines for Good Clinical Practice and the Declaration of Helsinki. All living patients provided written informed consent before enrollment. Informed consent was waived for dead patients before study initiation.

## Author contributions

UA-H: Conceptualization, Data curation, Formal analysis, Funding acquisition, Investigation, Methodology, Project administration, Resources, Software, Supervision, Validation, Visualization, Writing – original draft, Writing – review & editing. OF-C: Conceptualization, Supervision, Validation, Writing – review & editing. JR-B: Methodology, Supervision, Validation, Visualization, Writing – review & editing. SM-B: Supervision, Validation, Writing – review & editing. NF-N: Validation, Writing – review & editing. ZN-S: Validation, Writing – review & editing. MG-P: Validation, Writing – review & editing. JC-N: Validation, Writing – review & editing. GM-G: Validation, Writing – review & editing. PP-P: Validation, Writing – review & editing. AG-C: Validation, Writing – review & editing. DP-F: Validation, Writing – review & editing. LS-C: Validation, Writing – review & editing. ML: Supervision, Validation, Writing – review & editing. AM-D: Validation, Writing – review & editing. AM-C: Validation, Writing – review & editing. SV-E: Conceptualization, Funding acquisition, Methodology, Project administration, Supervision, Validation, Writing – review & editing.
