# Supplementary material for: Outcomes and patterns of use of Radium-223 in metastatic castration-resistant prostate cancer
Source: Front Oncol. 2024 May 7;14:1385466. doi: 10.3389/fonc.2024.1385466 (PMC11106362; doi:10.3389/fonc.2024.1385466)
Supplement: Supplementary file 1 [file DataSheet_1.docx]

Supplementary Material

# Supplementary Figures

## Supplementary Figures


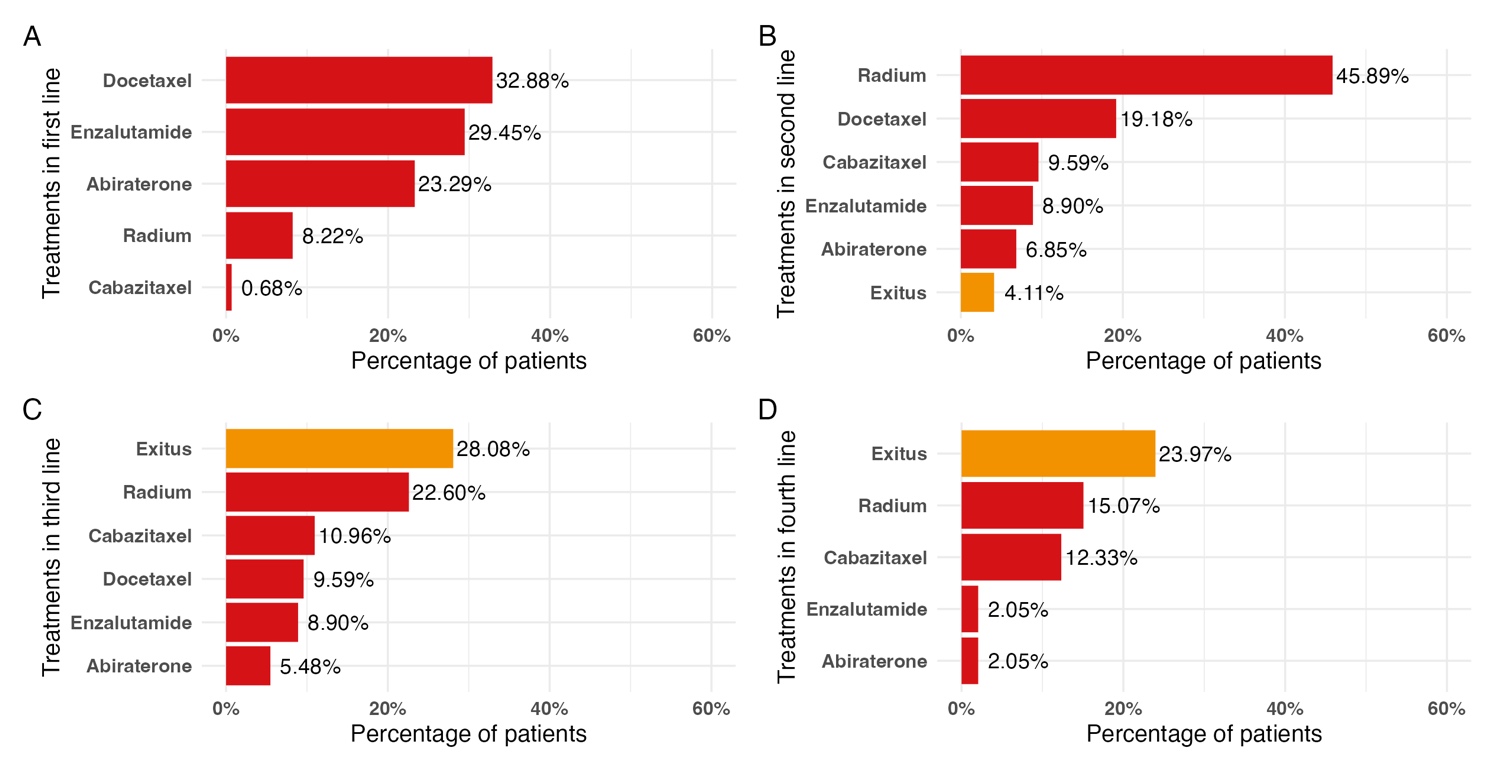


**Supplementary Figure 1.** Treatments used by line and percentage of patients who died without receiving treatment in that line. A, first line; B, second line; C, third line; D, fourth line.
